# Editorial: Role of hypoxia-inducible factors in metabolic immune cell adaptation during sepsis

**DOI:** 10.3389/fimmu.2023.1194504

**Published:** 2023-04-18

**Authors:** Carlos del Fresno, Leon Nicolas Schulte, Eduardo López-Collazo

**Affiliations:** ^1^ The Innate Immune Response Group, Hospital la Paz Institute for Health Research (IdiPAZ), La Paz University Hospital, Madrid, Spain; ^2^ Immunomodulation Lab, IdiPAZ, La Paz University Hospital, Madrid, Spain; ^3^ Institute for Lung Research, Faculty of Medicine, University of Marburg, Marburg, Germany; ^4^ German Center for Lung Research (DZL), Giessen, Germany; ^5^ Tumour Immunology Laboratory, IdiPAZ, La Paz University Hospital, Madrid, Spain

**Keywords:** HIF, sepsis, metabolism, myeloid cells, inflammation

## Unraveling the immunological complexity of sepsis

Sepsis is a life-threatening organ dysfunction caused by a dysregulated host response to infection that remains a leading cause of death in intensive care units worldwide. The global burden of sepsis is difficult to ascertain, the latest statistics report that in 2017 there were 48.9 million cases and 11 million sepsis-related deaths worldwide, which accounted for almost 20% of all global deaths (1).

Two phases have been recognized in this disease: an early inflammatory phase and a late immunosuppressive stage; however, these two stages can overlap. Several characteristic alterations have been described during sepsis including decreased human leukocyte antigen (HLA)-DR expression, overexpression of immune checkpoints, and T cell exhaustion. Monocytes/macrophages are believed to play an important role in this context by functioning as orchestrating hubs of the host immune response. They participate in both phases of sepsis, firstly by releasing inflammatory cytokines that contribute to inflammatory pathologies, and secondly by adopting an immune depressive phenotype, characterized by a diminished response to pathogen signatures, inflammatory stimuli, and, therefore, secondary infections. During the transition from a pro-inflammatory to an immunosuppressive phenotype, monocytes/macrophages adopt protective functions including increased phagocytosis, bactericidal activity, and tissue remodeling. Furthermore, they participate in the induction of T cell exhaustion through the expression of immune checkpoints. This reflects their functional plasticity during human sepsis (2).

The phenotypic switch of monocytes/macrophages during the course of sepsis is critically controlled by hypoxia-inducible factor-1α (HIF1α) expression. HIF1α is a major regulator of oxygen homeostasis in mammals. Under normoxia, oxygen and prolyl hydroxylases hydroxylate HIF1α, inducing its ubiquitination and further proteasomal degradation after binding of ubiquitin ligase proteins such as Von Hippel-Lindau (VHL) protein. During the inflammatory phase of sepsis, hypoxic conditions suppress hydroxylation of HIF1α resulting in its accumulation and nuclear translocation to activate critical metabolic adaptation pathways (3). In blood, an abnormally low level of oxygen is known as hypoxemia. How hypoxia and HIF1α driven metabolic adaptations affect the course of infections and sepsis outcome is incompletely understood. Whereas hypoxemia alone is considered a bad prognostic marker, an improved mechanistic understanding of hypoxia driven adaptations might reveal important cues linking immune status and outcome in sepsis patients.

## Multifaceted roles of HIFs in disease-associated cell compartments

Several studies in this collection examine the role of hypoxia-inducible transcription factors (HIFs) in disease, with a focus on sepsis. The reports suggest an important role for HIFs in controlling essential cell functions in the immune system and in immunoregulatory cells, but also point to limitations regarding future HIF-based therapies.

Vanderhaeghen et al. dissect the roles of HIF1α and HIF2α in mouse models of sepsis. They observed that expression of HIF1α and HIF2α is induced in liver tissue during polymicrobial sepsis. However, using knockout mice with liver-specific loss of HIF1α and HIF2α, they found no evidence for a contribution of either factor to survival in polymicrobial sepsis. The authors conclude that the contribution of hepatically expressed HIF1α and HIF2α to lethality in sepsis is minor. However, the specific depletion of HIF1α in myeloid cells shows a massive impact in LPS-induced sepsis, conferring protection by reducing the levels of circulating pro-inflammatory cytokines (4). Along the same line, the depletion of HIF1α in macrophages dampened their bactericidal capacity against Group A *Streptococcus*, rendering these mice more susceptible to this bacterial infection (5). Therefore, HIF proteins seem to have a more predominant role in hematopoietic than stromal cells under septic conditions. Nevertheless, it would be interesting to address the relative contribution of both compartments to the HIF expression observed by Vanderhaeghen et al. in total liver.

Since the immune response in sepsis overlaps to a large extent with immune reactions in other systemic diseases, it may be possible to learn from the role of HIFs in major diseases such as cancer or COPD for future sepsis therapies. In a study by Wang et al. the role of HIF1α in COPD is investigated - a disease mainly caused by tobacco smoke that, like sepsis, has a significant systemic component. Advanced COPD patients often suffer from reduced peripheral blood oxygen saturation. The authors report that in fibrocytes, which are mesenchymal progenitors that accumulate in diseased tissue, HIF1α is induced under hypoxic conditions and controls the expression of factors associated with fibrocytic differentiation and proliferation. Interestingly, adoptively transferred fibrocytes were previously reported to improve sepsis survival *via* modulation of T-cell activity (6). The role of HIF1α in fibrocyte differentiation, reported in this article collection, should therefore be considered in future cell-based sepsis therapies. In addition to the role of HIFs in fibrocytes, which have T cell modulating properties, this collection also points to a direct role of HIF1 in T cells. Bargiela et al. report a role of active vitamin B6 metabolism in CD8^+^ T cell proliferation and differentiation. They propose that HIF1 is a key regulator of vitamin B6 metabolism in T cells. The functional relevance of this regulation is shown by the necessity of vitamin B6 metabolism in CD8^+^ T-cell dependent antitumor immunity against mouse B16 melanoma. Given the diverse roles of CD8^+^ T cells and metabolic reprogramming in sepsis (7, 8), it may prove valuable to elucidate the role of the HIF1-vitamin B6 axis in this context.

## Sepsis markers linked to HIFs

The search of highly predictive risk factors for sepsis-associated mortality remains an ongoing challenge. Other studies in this collection set out to identify further sepsis markers and targets. Using diverse readouts, including flow-cytometry and RNA-seq, Lei et al. investigate peripheral blood markers associated with the development of sepsis associated delirium (SAD) and mortality. They report the CD14^hi^/CD16^-^ monocyte percentage to be reduced in peripheral blood from SAD patients. Furthermore, they find increased SLC2A1/GLUT1 and decreased STIMATE expression levels to be predictive of patient survival. Interestingly, SLC2A1 is a direct transcriptional target of HIF1α (3). In another biomarker study in this collection, Ming et al. present a bioinformatics driven approach to deduce biomarkers of sepsis-associated acute respiratory distress syndrome (ARDS), based on co-expression network analysis. ARDS is a particularly frequent complication during sepsis. The analysis performed by Ming et al. reveal SIGLEC9, TSPO, CKS1B and PTTG3P as biomarkers for the discrimination of sepsis-associated ARDS stages and for associated alterations in the peripheral immune cell compartment. Among these markers, the expression of TSPO and HIF1α were found to be correlated upon experimental conditions reducing cell proliferation (9), and PTTG3P expression was reported to depend on HIF1α under hypoxia (10). Therefore, HIF activation seems a promising driver of biomarkers associated with sepsis-related pathological conditions.

## Untangling the role of HIF in sepsis, friend or foe?

Based on the clinical results included in this collection, HIF activation seems to anticipate a worsened prognosis in COPD, sepsis related delirium (SAD) and acute respiratory distress syndrome (ARDS). To which extent this detrimental role is tied to an immunosuppressive or proinflammatory response is unclear. However, activation of HIF1α has been associated with the development of endotoxin tolerance, a systemic process observed in septic patients through which their myeloid cells show decreased cytokine production and higher phagocytic and tissue re-modeling capacity upon a secondary challenge (11). Yet, HIF1α has also been described as the driver of the glycolytic metabolic program underlying the induction of trained immunity, the process by which myeloid cells generate a boosted inflammatory response following a secondary insult (12). Therefore, the activation of the same transcription factor generates two apparently opposite inflammatory conditions. A deeper knowledge about HIFs in the septic context could untangle this controversy.

Taken together, the publications in this collection, based on studies both using animal models and clinical observations, are advancing our understanding of the role of HIFs and additional factors in sepsis and other diseases. Further studies may build on the findings presented here to achieve much-needed new treatment regimens for critically ill patients.

## Author contributions

All authors listed have made a substantial, direct, and intellectual contribution to the work and approved it for publication.
